# CRISPR/Cas9-generated mouse model with humanizing single-base substitution in the *Gnao1* for safety studies of RNA therapeutics

**DOI:** 10.3389/fgeed.2023.1034720

**Published:** 2023-04-03

**Authors:** Anna V. Polikarpova, Tatiana V. Egorova, Evgenii A. Lunev, Alexandra A. Tsitrina, Svetlana G. Vassilieva, Irina M. Savchenko, Yuliya Y. Silaeva, Alexey V. Deykin, Maryana V. Bardina

**Affiliations:** ^1^ Laboratory of Modeling and Gene Therapy of Hereditary Diseases, Institute of Gene Biology Russian Academy of Sciences, Moscow, Russia; ^2^ Marlin Biotech, Sochi, Russia; ^3^ Center for Precision Genome Editing and Genetic Technologies for Biomedicine, Institute of Gene Biology, Russian Academy of Sciences, Moscow, Russia; ^4^ Koltzov Institute of Developmental Biology Russian Academy of Sciences, Moscow, Russia; ^5^ Core Facility Center, Institute of Gene Biology Russian Academy of Sciences, Moscow, Russia; ^6^ Laboratory of Genetic Technologies and Genome Editing for Biomedicine and Animal Health, Joint Center for Genetic Technologies, Belgorod National Research University, Belgorod, Russia

**Keywords:** GNAO1 encephalopathy, genetically modified mice, CRISPR/Cas9, genome editing, personalized medicine

## Abstract

The development of personalized medicine for genetic diseases requires preclinical testing in the appropriate animal models. GNAO1 encephalopathy is a severe neurodevelopmental disorder caused by heterozygous *de novo* mutations in the *GNAO1* gene. *GNAO1* c.607 G>A is one of the most common pathogenic variants, and the mutant protein Gαo-G203R likely adversely affects neuronal signaling. As an innovative approach, sequence-specific RNA-based therapeutics such as antisense oligonucleotides or effectors of RNA interference are potentially applicable for selective suppression of the mutant *GNAO1* transcript. While *in vitro* validation can be performed in patient-derived cells, a humanized mouse model to rule out the safety of RNA therapeutics is currently lacking. In the present work, we employed CRISPR/Cas9 technology to introduce a single-base substitution into exon 6 of the *Gnao1* to replace the murine Gly203-coding triplet (GGG) with the codon used in the human gene (GGA). We verified that genome-editing did not interfere with the Gnao1 mRNA or Gαo protein synthesis and did not alter localization of the protein in the brain structures. The analysis of blastocysts revealed the off-target activity of the CRISPR/Cas9 complexes; however, no modifications of the predicted off-target sites were detected in the founder mouse. Histological staining confirmed the absence of abnormal changes in the brain of genome-edited mice. The created mouse model with the “humanized” fragment of the endogenous *Gnao1* is suitable to rule out unintended targeting of the wild-type allele by RNA therapeutics directed at lowering *GNAO1* c.607 G>A transcripts.

## Introduction

The *GNAO1* gene (Gene ID: 2775) is abundantly expressed in neurons and conserved across multiple vertebrate (chimpanzee, rhesus monkey, dog, cow, mouse, rat, chicken, zebrafish) and invertebrate (fruit fly, *C. elegans*) species. The *GNAO1* encodes the Gαo subunit of the heterotrimeric G proteins which plays a key role in neuronal signal transduction in the brain (Jiang and Bajpayee, 2009). The *GNAO1* gene and Gαo protein have become a focus of intensive research following the discovery in 2013–2014 that its mutations are linked to the neurodevelopmental disorder currently termed the GNAO1 encephalopathy (Epi4K Consortium et al., 2013; Nakamura et al., 2013). The *GNAO1* mutations and dysregulated expression levels are also associated with malignant cell transformation and tumorigenesis (Liu et al., 2014; Xu et al., 2018; Song et al., 2021).

GNAO1 encephalopathy is a severe neurological disease that manifests in infants or young children and is caused by heterozygous *de novo* mutations in the *GNAO1* gene. Roughly 200 patients have been reported to date (The Bow Foundation, 2022) and 25 clinical variants of *GNAO1* are described in the literature (Feng et al., 2018). *GNAO1* mutations are characterized by clinical heterogeneity and present as movement disorder (OMIM 617493) or early onset epileptic encephalopathy (OMIM 615473) (Saitsu et al., 2016; Kelly et al., 2019). According to several studies, such heterogeneity is related to how different mutations affect the functioning of the Gαo protein in neurons. Feng et al. (2017) demonstrated in the culture-based assay that pathogenic mutations can decrease (loss-of-function) or enhance (gain-of-function) the ability of Gαo to inhibit cAMP production. Solis and Katanaev (2017) speculated that the cause of the disease depends on a particular mutation affecting Gαo function at the plasma membrane or the Golgi apparatus. The most recent study made an intriguing observation that different clinical mutations in Gαo interfere with the processing of neuromodulatory signals in a neuron-type-specific manner (Muntean et al., 2021). Phenotypic heterogeneity determines the need for personalized medicine for individual clinical variants of *GNAO1* and the creation of the appropriate animal models for preclinical testing.

An interesting case is missense variant *GNAO1* c.607 G>A (polymorphism rs587777057) which accounts for ∼15% of GNAO1-related disorder cases (Arya et al., 2017; Axeen et al., 2021). Patients with c.607 G>A mutation develop both epileptic seizures and movement disorder (Arya et al., 2017). Mutation results in p. Gly203Arg (G203R) substitution near the key catalytic site of the Gαo, and presumably disrupts GTP binding which regulates the activity of the subunit (Nakamura et al., 2013). The exact mechanism by which mutant Gαo alters downstream signaling is an area of intensive research (Feng et al., 2017; Mihalek et al., 2017; Muntean et al., 2021; Wang et al., 2021). Different groups obtained conflicting results and characterized *GNAO1* c.607 G>A mutation as gain-of-function (Feng et al., 2017) or loss-of-function with a dominant negative effect (Muntean et al., 2021; Wang et al., 2021) or as a neomorphic mutation (Larasati et al., 2022). Nevertheless, the authors agree that the functional activity of Gαo-G203R impairs neuronal signaling and is the primary cause of the pathological condition.

Given the functional characterization of the clinical variant *GNAO1* c.607 G>A, innovative RNA-based therapies that selectively suppress the expression of the mutant transcript might provide therapeutic benefits. RNA-directed therapeutics are sequence-specific (Zhu et al., 2022; Zogg et al., 2022), and two different classes of such drugs are currently in development for neurological disorders: antisense nucleotides (ASO) that downregulate transcript *via* RNase H recruitment and adeno-associated vector (AAV)-based RNA interference (RNAi) technology (AAV-RNAi) (Schoch and Miller, 2017; Martier and Konstantinova, 2020; Helm et al., 2022). In the case of GNAO1 encephalopathy, transcript with c.607 G>A substitution should be silenced in an allele-specific manner without affecting the wild-type allele. Common approach to achieve allele selectivity is to design RNA-suppressing therapeutics complementary to the short fragment (<25 nucleotides) of the mRNA encompassing pathogenic mutation, which discriminates the mutant variant from the wild-type allele (Sibley and Wood, 2011; Jiang et al., 2013; Luo et al., 2018; Morelli et al., 2019; Helm et al., 2022).

Available models for *in vitro* proof-of-concept studies of RNA therapeutics for GNAO1 encephalopathy are patient-specific neurons and organoids derived from induced pluripotent stem cells (Akamine et al., 2020). To evaluate drug efficacy *in vivo*, a mouse model with a heterozygous c.607 G>A mutation in murine *Gnao1* is required. Despite efforts of independent research groups, no mouse line bearing c.607 G>A mutation was established due to the severe neonatal lethality (Feng et al., 2019; Silachev et al., 2022). Moreover, RNA-based therapeutics such as ASO and AAV-RNAi may have toxicity arising from incomplete allele-selectivity and downregulation of the wild-type protein (Schoch and Miller, 2017). To study *in vivo* whether the RNA therapeutics directed at *GNAO1* c.607 G>A also suppress the wild-type transcript, a mouse model with the humanized endogenous *Gnao1* is required. While full humanization of the gene-of-interest is aimed (Zhu et al., 2019), it is beneficial to replace with human sequence a gene fragment directly targeted by RNA therapeutics (typically a window of ±20–30 nucleotides around the mutation site).

In this study, we applied CRISPR/Cas9 technology to generate a mouse line with the sequence of the wild-type Gnao1 encompassing rs587777057 identical to the human gene. For rapid identification of the genome-edited animals, we introduced the restriction site into the intronic region and developed TaqMan qPCR to detect wild-type murine and “humanized” *Gnao1* alleles. Expression level of the “humanized” *Gnao1* mRNA and the encoded Gαo protein didn’t differ from the wild-type mice; the localization of Gαo in the brain tissues was also not affected. Neither off-target genome modification nor histological evidence of adverse events was present in the brain of genome-edited mice. We conclude that the novel mouse line meets the criteria to address unintended silencing of the wild-type allele by RNA therapeutics directed to clinical variant *GNAO1* c.607 G>A.

## Materials and methods

### Animals

Animal studies were carried out in compliance with Directive 2010/63/EU. All experimental protocols were approved by the local Ethics Committee of the IGB RAS. (C57BL/6xCBA/lac) F1 female and male mice for oocyte generation and CBA mice for breeding were purchased from vivarium “Stolbovaya” (Russia).

### CRISPR target sequence design

Guide sequence for CRISPR/Cas9 to target *Gnao1* was designed manually. The proto-spacer adjacent motif (PAM) sequence (5′-NGG-3′) was chosen in the intronic sequence of *Gnao1* in the proximity to the G203-coding triplet located in exon 6. The 19 nucleotides upstream of the PAM site (5′-GCT​TTC​CCT​GAC​TCC​CTG​C-3′) were selected as a sgRNA targeting sequence (Bardina et al., 2021).

### Preparation of Cas9 mRNA and sgRNA

Cas9 mRNA and sgRNAs were prepared by *in vitro* T7 transcription as previously described (Egorova et al., 2019). mRNA for Cas9 nuclease from *S. pyogenes* was synthesized using the mMESSAGE mMACHINE T7 Kit (Ambion, Japan) on a template of linearized pET28a/Cas9-Cys construct from Hyongbum Kim Lab (Addgene plasmid #53261). mRNA was purified from the reaction mix by phenol-chloroform extraction followed by isopropanol precipitation. Air-dried RNA was resuspended in nuclease-free water and concentration was determined with the Qubit RNA HS kit (Thermo Fisher Scientific). RNA stock was diluted to 500 ng/uL, aliquoted, and stored at − 70°C before use.

To synthesize sgRNAs, a DNA amplicon was generated by overlapping PCR with forward (5′-GAA ATT AAT ACG ACT CAC TAT AGG GCT TTC CCT GAC TCC CTG CGT TTT AGA GCT AGA AAT AGC-3′) and reverse (5′-AAA AGC ACC GAC TCG GTG CCA CTT TTT CAA GTT GAT AAC GGA CTA GCC TTA TTT TAA CTT GCT ATT TCT AGC TCT AAA AC-3′) oligos. Uniquely designed forward oligo contains T7 promoter, transcription initiation site, *Gnao1* targeting sequence (5′-GCT​TTC​CCT​GAC​TCC​CTG​C-3′), and the scaffold-specific sequence; standard reverse oligo includes the remaining portion of the sgRNA scaffold. Oligos were combined in an equimolar ratio (1 uM each) and amplified using Pfu DNA polymerase (Promega, M7745): 30 cycles of 95°C for 30 s, 65°C for 30 s, 72°C for 30 s. The amplicon was cleaned up from the reaction mix and *in vitro* transcribed using the RiboMAX Express kit (Promega, P1320). sgRNA was extracted with TRIzol Reagent (Thermo Fisher Scientific, 15596026) followed by isopropanol precipitation. sgRNA was dissolved in nuclease-free water to 250 ng/uL, aliquoted, and stored at − 70°C before use.

### ssODN repair template

Single-stranded DNA oligonucleotide (ssODN+, 90 nt) 5′-TCT AGC TCT TAG GCG TCC CCG CCC TCA CAG CTT TCC CTG ACT ACC TGC AGG CTG TTT GAC GTC GGA GGC CAG CGA TCT GAA CGC AAG AAG-3’ (Bardina et al., 2021) was chemically synthesized and desalted by Evrogen (Russia). Lyophilized oligo was resuspended in sterile water to 100uM and stored at −20°C.

### Microinjection into mouse zygotes and embryo transfer

Superovulation was induced in (C57BL/6xCBA/lac) F1 females weighing 12–13 g by intraperitoneal injection of pregnant mare serum gonadotropin (Folligon, Intervet International, 5 units/mouse) followed by human chorionic gonadotropin (hCG, Pregnil, N.V. Organon, 5 units/mouse) in 46–48 h. After injections, the female mice were mated with (C57BL/6xCBA/lac) F1 males. Fertilized eggs were surgically washed out 12–13 h after copulation and visually inspected. The two-pronuclear zygotes were transferred to a depression slide in an M2 medium (MTI-GlobalStem, United States) overlaid with embryo-safe mineral oil (Merck KGaA, Germany) (Zvezdova et al., 2010). 50 ng/uL Cas9 mRNA, 18.6 ng/uL sgRNA, and 10 uM ssODN were mixed in the injection buffer (10 mM Tris-HCl pH 7.4; 0.1 mM EDTA) and diluted 2- and 5-fold in the same buffer in the set of the optimization experiments. The injection mix was incubated for 5 min at 65°С followed by centrifugation at 14,000 rpm for 5 min. Microinjection of the Cas9 mRNA/sgRNA/ssODN mix was performed into the cytoplasm and male pronuclei of one-cell-stage zygotes using differential interference contrast microscope Axiovert 200 (Carl Zeiss, Germany) equipped with a micromanipulator. After microinjection, the zygotes were cultured for 2–3 h at 37°C in an atmosphere of 5% CO2 and then assessed visually. For blastocyst assay, the zygotes were transferred into 35 mm Petri dishes and cultured in 50–60 μL droplets of KSOM medium (MTI-GlobalStem) under mineral oil at 37°C and 5% CO2 for 3 days till the blastocyst stage. To produce genome-modified mice, injected eggs were incubated overnight, and viable two-cell-stage embryos were implanted into pseudopregnant foster dams (Silaeva et al., 2018).

### Blastocyst assay

Single blastocyst assay (Sakurai et al., 2014) was performed with modifications described in (Dimitrieva et al., 2016). To prepare crude DNA, a single blastocyst was washed briefly in nuclease-free water, transferred into a PCR tube, and lysed in 20 μL of blastocyst lysis buffer (100 mM Tris-HCl, pH 8.3, 100 mM KCl, 0.02% gelatin, 0.45% Tween 20, 125 μg/mL Proteinase K) for 10 min at 56°C followed by 10 min inactivation at 95°C. The resulting blastocyst-derived DNA solution was stored at −20°C until use. For analysis, 5 μL of crude DNA was used in a 20 μL PCR reaction to amplify the *Gnao1* genomic region with the forward (5′-ACT​AGG​AGA​CGG​AGA​GGT​GAG-3′) and reverse (5′-GTG​CGT​CCT​AGC​CAA​GAC​C-3′) primers under the following conditions: 95°C for 5 min, 40 cycles at 95°C for 30 s, 60°C for 30 s, 72°C for 1 min; 72°C for 5 min. The resulting amplicons (533 bp) were detected by electrophoresis in a 2% agarose gel and purified for Sanger sequencing with the forward primer. Sequencing reads were checked in Geneious Prime Software and analyzed for indel/knock-in efficiency using the online tool “Synthego” (Synthego - CRISPR Performance Analysis).

### Breeding and genotyping

Female founder mouse with edited *Gnao1* allele was crossed against CBA male mouse to generate heterozygous F1 offspring. Further breeding was done to produce homozygous and heterozygous F2 backcrosses that were subjected to genotyping. Mouse tail biopsies were collected at the age of 1–2 weeks and incubated in an alkaline lysis buffer [25 mM NaOH, 0.2 mM Na2-EDTA (pH 12.0)] for 1 h at 95°C followed by neutralization with 40 mM Tris-HCl pH 5.0. The resultant crude genomic DNA solution (2 μL) was used in PCR reaction with primers and conditions described above for blastocyst assay. For genotyping by restriction assay, 250 ng of purified 533 bp-long amplicons were incubated with 0.5 units of BspMI enzyme (New England Biolabs, R0502S) in 1X NEBuffer 3.1 for 1 h at 37°C. DNA restriction fragments were analyzed by electrophoresis in 2% agarose gel. For genotyping by allelic qPCR, 2 μL of crude DNA was used in a 25 μL reaction mix containing qPCR master mix (M-428, Syntol, Russia), PCR stabilizer (B023, SibEnzyme, Russia), 400 nM primers (Gnao1-DNA-F: 5′-CTC​ACT​CTC​ACC​TCT​AGC​TCT​T-3′, Gnao1-R: 5′-GGA​TCC​ACT​TCT​TGC​GTT​CA-3′), 200 nM TaqMan probes for single nucleotide polymorphisms (SNPs) or total *Gnao1* detection. Probes specific to mouse *Gnao1* (Gnao1-ROX: ROX-CGTCGGGGGCCAGC-BHQ2) and for SNP-irrespective *Gnao1* detection (Gnao1-DNA-Cy5: FAM-AGTCAGGGAAAGCTGTGAGGGC-BHQ1) were designed using PrimerQuest Tool from Integrated DNA Technologies (IDT), probe to “humanized” *Gnao1* (hGnao1-FAM: FAM-CGTCGGAGGCCAGC-BHQ1 (Lunev et al., 2022a), was synthesized by DNA-Synthesis (www.oligos.ru, Moscow, Russia). Allelic PCR reaction was performed at 95°C for 3 min, 40 cycles at 95°C for 15 s, 58°C for 20 s, and 72°C for 30 s using CFX96 Touch Real-Time PCR Detection System (Bio-Rad Laboratories, United States). Genotype was assigned based on amplification in FAM or ROX fluorescent channels. The genotype of selected mice was verified by Sanger sequencing of 533 bp amplicons. A colony of genome-edited mice was produced on the background of CBA mice and referred to as Gnao1-GGA. The *Gnao1* expression and off-target studies were performed on F2 and F3 mice in comparison with the wild-type littermate controls.

### Brain samples collection

10–12 weeks old mice were anesthetized with an overdose of Zoletil 100 (Virbaс, France) and Xyla (Interchemie, Netherlands), and brains were rapidly removed from the skull. For expression studies, brain tissues were collected and snap-frozen in liquid nitrogen. Following grinding, 20 mg aliquots of powdered tissues were made and further processed for RNA extraction or protein lysate preparation. For immunofluorescence staining, whole brains were fixed for 12 h in 4% paraformaldehyde in PBS at 4°C. Fixed brains were transferred to 30% sucrose and incubated at 4°C until the brains sank to the bottom (Kim et al., 2014). Cryoprotected brains were immobilized on Tissue-Tek O.C.T. Compound (Sakura Finetek) and frozen on dry ice.

### RNA analysis

RNA from powdered brain tissues was isolated using TRIzol Reagent (Thermo Fisher Scientific, 15596026) supplemented with 1-bromo-3-chloropropane (Sigma, B9673) following the manufacturer’s protocol. RNA concentration was measured, and 2 µg of RNA was treated with DNase I, RNase-free (Thermo Fisher Scientific, EN0521). Reverse transcription performed using MMLV RT kit (SK021, Evrogen) with a mixture of 1uM random (dN)10 (SB002, Evrogen) and 1uM oligo (dT)15 (SB001, Evrogen) primers.

For Sanger sequencing, the *Gnao1* region including the exon 6 was amplified with forward (Gnao1-cDNA-F: 5′-TAC​TAC​CTG​GAC​AGC​CTG​GA-3′) and reverse (Gnao1-R: 5′-GGA​TCC​ACT​TCT​TGC​GTT​CA-3′) primers. The resulting amplicons (178 bp) were detected by electrophoresis in a 2% agarose gel and purified for Sanger sequencing with the reverse primer. Sequencing reads were analyzed with the Geneious Prime Software.

For mRNA expression analysis, primers, TaqMan probes, and qPCR reaction conditions for mouse *Gnao1* and *Gapdh* were previously described (Lunev et al., 2022b). Here, hGnao1-FAM probe specific to the “humanized” *Gnao1* (see above) and probe for total *Gnao1* mRNA detection (Gnao1-RNA-Cy5: Cy5-TGGCATCGTAGAAACCCACTTCACC-BHQ2) were added to the multiplex reaction. Another multiplex contained primers/probes to detect the expression of the housekeeping genes Ap3d1 and Csnk2a2 (Hildyard et al., 2019) as previously described (Starikova et al., 2022). All reactions were carried out in CFX96 TOUCH Real-Time PCR Detection System (Bio-Rad Laboratories). The copy number of *Gnao1* mRNA per 100 ng of total RNA in the brain samples was calculated based on the standard curves built with serial dilutions of the reference plasmids containing the CDS of murine *Gnao1* (pMusGNAO1) or human *GNAO1* (pGNAO1) (Lunev et al., 2022b). *Gnao1* transcript copy number was normalized to the expression of the housekeeping genes *Gapdh*, *Ap3d1*, and *Csnk2a2*.

For the analysis of splice variants, the *Gnao1* region was amplified with the forward (Gnao1-cDNA-F) and reverse (Gnao1-cDNA-R: 5′-TCT​TTG​TTG​GGT​GAG​CGG​TT-3′) primers spanning from exon 5 to 8. PCR was performed with the following conditions: 98°C for 3 min, 30 cycles at 98°C for 10 s, 65°C for 15 s, 72°C for 15 s; 72°C for 5 min. The resulting amplicons (491 bp) were detected by electrophoresis in a 2% agarose gel.

### Western blotting

Protein samples were prepared by lysing 20 mg of powdered tissue in the buffer: 50 mM Tris-HCl pH 7,8, 150 mM NaCl, 1% SDS, 1 mM EDTA, and protease inhibitor cocktail (Roche, Cat #1873580). The lysate was clarified by centrifugation at 14,000 g for 15 min. Protein concentration was measured using Quick Start Bradford 1x Dye Reagent (Bio-Rad, Cat #500–0205) and QuickStart BSA Standard Set (Bio-Rad, Cat #500–0207), readings were done on CLARIOstar Plus (BMG Labtech). Each protein lysate (10 μg or 5 μg for the abundance) was resolved in 12% SDS-polyacrylamide electrophoresis gel and transferred to a nitrocellulose membrane using the Trans-Blot Turbo Blotting System (Bio-Rad). The membrane was blocked in 5% dry milk in TBS-T and incubated for 1 h at room temperature with antibodies diluted in blocking solution: anti-Gαo (rabbit; Thermo Fisher Scientific, PA5-30044; 1:5,000 dilution), anti-GAPDH (mouse; Sigma-Aldrich, G8795; 1:20,000), anti-beta III Tubulin (rabbit; Abcam, ab18207; 1:1,000). Following washes in TBS-T the appropriate HRP-conjugated secondary antibodies were used: anti-rabbit (Bio-Rad, Cat# 170–6515; 1:3,000) and anti-mouse (Bio-Rad, Cat# 170–6516; 1:3,000). Proteins were detected using Clarity Western ECL substrate (BioRad, #170–5,060) and iBright 1500 Imaging System (Thermo Fisher Scientific).

### Immunohistochemistry

20 μm cryosections of the murine brains were prepared using Leica CM 1510–1 Cryostat Microtome, fixed in 4% PFA in PBS and permeabilized with 0.1% Triton X-100 in PBS. Sections were blocked with 5% bovine serum albumin and 0.1% Triton X-100 in PBS solution, stained overnight with anti-Gαo antibody (rabbit; Thermo Fisher Scientific, PA5-30044; 1:300 dilution), followed by staining for 1 h with Alexa Fluor 633 anti-rabbit secondary antibody (Invitrogen, A21072; 1:1,000) and nuclei counterstaining with DAPI dye (1:1,000) for 30 min. Antibodies and DAPI dye were diluted in the blocking buffer. Three 15 min washes with 0.1% Triton X-100 in PBS were included after each step. Images were acquired using Zeiss LSM880 confocal microscope equipped with Plan-Apochromat 20x/0.8 M27 objective.

### Histological analysis

For Hematoxylin/Eosin (H&E) and Nissl staining, whole brain samples were fixed in 10% buffered formalin and embedded in paraffin. Frontal and sagittal sections (10 μm thick) were stained with H&E and Nissl according to standard procedure (Slaoui et al., 2017). Images of stained sections were acquired with a Keyence BZ-X710 microscope.

### Off-target analysis

Potential off-target sites for the sgRNA in the mouse genome were predicted using the online E-CRISP Evaluation tool (Heigwer et al., 2014). The following parameters were set: number of 5′mismatch positions ignored by the program - 1, tolerated edit distance to the target sequence - 2 or 1. The latter stringent criteria were chosen to pick top candidate sites with minimum mismatches to the guide sequence and with canonical (NGG) or non-canonical (NAG) PAM. Top three gene targets were experimentally verified. Blastocysts and biopsies from CRISPR/Cas9-edited mice were handled and used for genomic DNA extraction as described above. Genomic regions surrounding off-target sites were amplified using primer pairs for *Tmem* (5′-TTT​GGG​GAC​ATA​AGC​AGG​CT-3′, 5′-CAA​TCG​CAG​GGC​AGA​TCC​T-3′), *Aurkaip1* (5′-CCC​AGG​AAG​ATG​GCC​ATC​AG-3′, 5′-CTT​CAA​ACG​TCC​TTC​CCG​GA-3′), and ChrX (5′-TGA​CAT​CTC​TCT​GCA​TGC​AAG​T-3′, 5′-TGT​CCA​CAT​GCT​ACA​TTG​ATT​GC-3′). Amplicons were analyzed in agarose gel, and bands were sequenced with the forward primer. Sequences were processed in Geneious 8.1.3, and the alternative alleles were determined using the Poly Peak Parser tool (Hill et al., 2014).

## Results

### Single-base substitution in *Gnao1* exon 6 using CRISPR/Cas9

**FIGURE 1:**
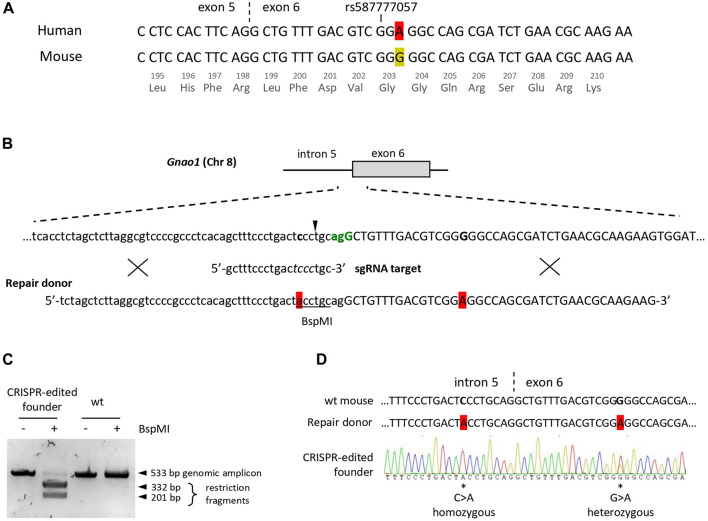
Creation of the founder mouse with the “humanized” fragment of the *Gnao1* using CRISPR/Cas9. **(A)** Sequence alignment of the human (NM_020988.3) and the mouse (NM_010308.3) *Gnao1* transcripts at the exon 5 - exon 6 junction (marked with dash line), ±25 nucleotides around the human polymorphism rs587777057 are shown. A single synonymous variation in Gly203-coding triplet is highlighted in red for A and yellow for G in human and mouse transcripts, respectively. **(B)** Graphical representation of the Cas9/sgRNA-assisted homologous recombination in mouse *Gnao1* gene using ssODN+ as a repair template. The genomic sequence of the intron 5 (lowercase) and exon 6 (uppercase) junction is shown. The PAM site for Cas9 (*S.pyogenes*) is in green and coincides with the 3′ splice site. An arrowhead indicates Cas9 cut site (3-nt upstream of the PAM site). Below is the sgRNA target sequence; mismatch-sensitive “core” sequence (positions +4 to +7 upstream of PAM according to (Zheng et al., 2017) in italic. Two nucleotides subjected to substitution in the *Gnao1* genomic sequence are in bold. ssODN+ (90 nt) is with two single-base substitutions highlighted in red, BspMI site is underlined. Crosses schematically demonstrate putative homology-directed repair. **(C)** Genotyping of the CRISPR/Cas9-edited founder mouse at P10 by restriction analysis. The genomic DNA fragment (533 bp) encompassing the Cas9 cleavage site was amplified from the tissue biopsy. Amplicon digestion with BspMI confirms the presence of ssODN-mediated knock-in in the mouse genome. **(D)** Sanger sequencing chromatogram of genomic DNA from the CRISPR/Cas9-edited founder mouse. Two single-base substitutions are marked by asterisks and their position in the genome is specified. Sequences of the wt mouse and ssODN are shown for reference.

To determine substitutions required for humanization of the endogenous *Gnao1* sequence near rs587777057, we aligned sequences of mouse and human transcripts at the exon 5-exon 6 junction (Figure 1A). Given that *Gnao1* is highly conserved across species (Krishnan et al., 2015), the mouse transcript contains a single synonymous variation as compared to the human transcript. The variable nucleotide is in the third position of the G203-coding triplet, namely, murine glycine is encoded by GGG in contrast to GGA in human mRNA. To “humanize” the selected *Gnao1* fragment, it is sufficient to introduce a single-nucleotide substitution G>A into exon 6, which will not affect the amino acid sequence of murine Gαo protein.

To generate genome-edited mice, we opted for knock-in technology including a CRISPR-Cas9 system (Figure 1B). As a repair template, we used single-stranded oligodeoxyribonucleotide (ssODN), an optimal donor for homology-directed repair and single-base substitutions (Ma et al., 2017; Okamoto et al., 2019). To increase the efficiency of genome editing, blocking mutations are frequently introduced to the proto-spacer adjacent motif (PAM) to abolish the re-cutting of the genomic DNA following the knock-in event. However, a blocking mutation in the coding region of *Gnao1* is inadmissible, so as not to сreate a mismatch between mouse and human sequences. Therefore, single-guide RNA (sgRNA) was designed to utilize the closest PAM in intron 5 which is also a 3′ splice site with AG/G consensus (Figure 1B). To reduce Cas9 re-cutting activity, additional single-base substitution C>A was introduced into a mismatch-sensitive “core” sequence of sgRNA (Zheng et al., 2017). This substitution also creates a BspMI restriction site in intron 5 useful for rapid genotyping. To knock in both single-nucleotide substitutions located 22 nucleotides apart (C>A in intron 5 and G>A in exon 6), we used 90 nt long ssODN as a donor template for the homology-directed repair (Figure 1B). Cleavage and knock-in efficiency achieved with genome editing components at various concentrations were evaluated by a single blastocyst-based assay (Supplementary Figure S1). Knock-in efficiency for *Gnao1* varied around 20%–25% (Supplementary Figure S1B) as compared to 30%–40% reported in a study using similar technology (Raveux et al., 2017). The optimal conditions (50 ng/uL Cas9 mRNA, 18.6 ng/uL sgRNA, 10 uM ssODN) were selected for further experiments.

A total of 569 zygotes were microinjected with CRISPR/Cas9 editing components and transferred to 74 recipients yielding five live-born potential founders. Only 0.2% (1 out of 569) of CRISPR-treated and transferred zygotes resulted in a single knock-in founder. Edited genomic DNA was detected in a female pup as early as at P0 by BspMI restriction analysis of the placenta material (Supplementary Figure S2). Genotyping by restriction and sequencing of the finger tissue obtained from P10 pups confirmed the presence of homozygous BspMI restriction site and heterozygous G>A knock-in in the *Gnao1* exon 6 (Figures 1C, D). The mosaic genomic sequence of the founder (Figure 1D) is probably due to the independent editing events of both alleles in the cell that gave rise to an embryo. The intronic mutation C>A incorporated more frequently in agreement with short (<5 nt) cut-to-mutation distance as compared to G>A substitution located 20 nucleotides from the CRISPR/Cas9 cleavage site (Paquet et al., 2016). Interestingly, no live F0 mice with indels were recovered. This could indicate the embryonic lethality resulting from *Gnao1* manipulation. Indeed, complete preweaning lethality was reported for the homozygous *Gnao1* knockout mouse strains generated *via* embryonic stem cell-based technology; heterozygous Gαo-deficient mice presented pathogenic phenotypes in several physiological systems (Jiang et al., 1998; International Mouse Phenotyping Consortium).

**FIGURE 2:**
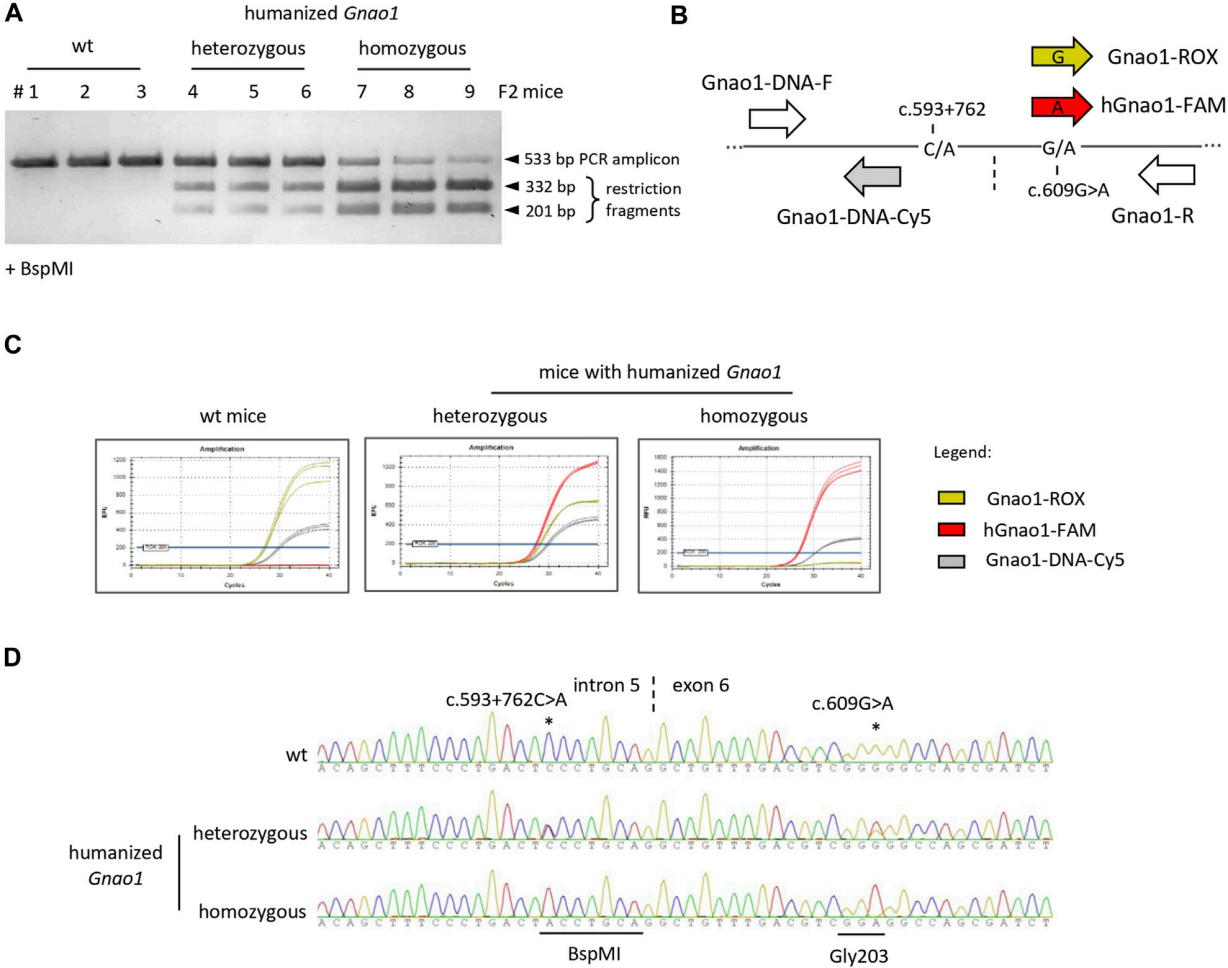
Genotyping of F2 littermates with single-base substitutions in *Gnao1*. **(A)** BspMI restriction analysis of genomic DNA amplicon (533bp). The traces of the uncut amplicon for the homozygous mice (lanes 7–9) are due to insufficient restriction enzyme digestion. **(B)** Schematically shown the position of primers and probes along the *Gnao1* genomic sequence used in allelic qPCR. Gnao1-ROX probe allows detection of the wild-type mouse *Gnao1* (GGG triplet for Gly203), hGnao1-FAM is specific for humanized *Gnao1* (GGA), Gnao1-DNA-Cy5 probe detects both *Gnao1* variants. Vertical dash line marks intron 5-exon 6 junction; **(C)** Genotyping by allele-specific TaqMan qPCR. Representative amplification curves demonstrate the selectivity of probes. **(D)** Confirming sequence of the *Gnao1* region by Sanger sequencing. Representative chromatograms are shown for wt, heterozygous, and homozygous genome-edited animals present in the same litter.

To establish a colony, the F2 litter of the founder mouse was genotyped using three independent approaches: restriction analysis, allele-specific qPCR, and Sanger sequencing (Figure 2). BspMI restriction was used as a fast and cost-effective method to screen the litter for the animals with knock-ins (Figure 2A). To detect variable nucleotides in the G203-coding triplet, we developed TaqMan probes that B discriminate G/A single-nucleotide polymorphism (SNP) (Figure 2B) and performed genotyping of the selected animals by allele-specific qPCR (Figure 2C). Finally, the sequence of the *Gnao1* genomic region surrounding rs587777057 was verified by Sanger sequencing (Figure 2D). Homozygous offspring were crossed on the CBA background to obtain a colony of mice with the “humanized” fragment of the endogenous *Gnao1* (*hGnao1*). These mice contain two single-base targeted mutations in the *Gnao1* gene: c.593 + 762C>A mutation creates BspMI site in the intron 5 and c.609G>A substitutes Gly203-coding triplet present in *Gnao1* (GGG) for GGA used in the human gene version. Following MGI Guidelines for nomenclature (2016, 2018), the newly engineered mouse strain was designated CBA.Cg-*Gnao1 ^em1(GNAO1)IGB^* (Bardina et al., 2021) and referred to as Gnao1-GGA mice further in the text.

### *Gnao1* expression and Gαo localization are not affected in genome-edited mice

To ascertain that genome editing didn’t disrupt the expression of the target gene, we examined the synthesis of the *Gnao1* mRNA and Gαo protein in Gnao1-GGA mice. By Sanger sequencing, we confirmed that mouse *Gnao1* transcript with c.609G>A substitution is produced, and its sequence is identical to its human counterpart over 68 nucleotides around rs587777057 (Supplementary Figure S3).

**FIGURE 3:**
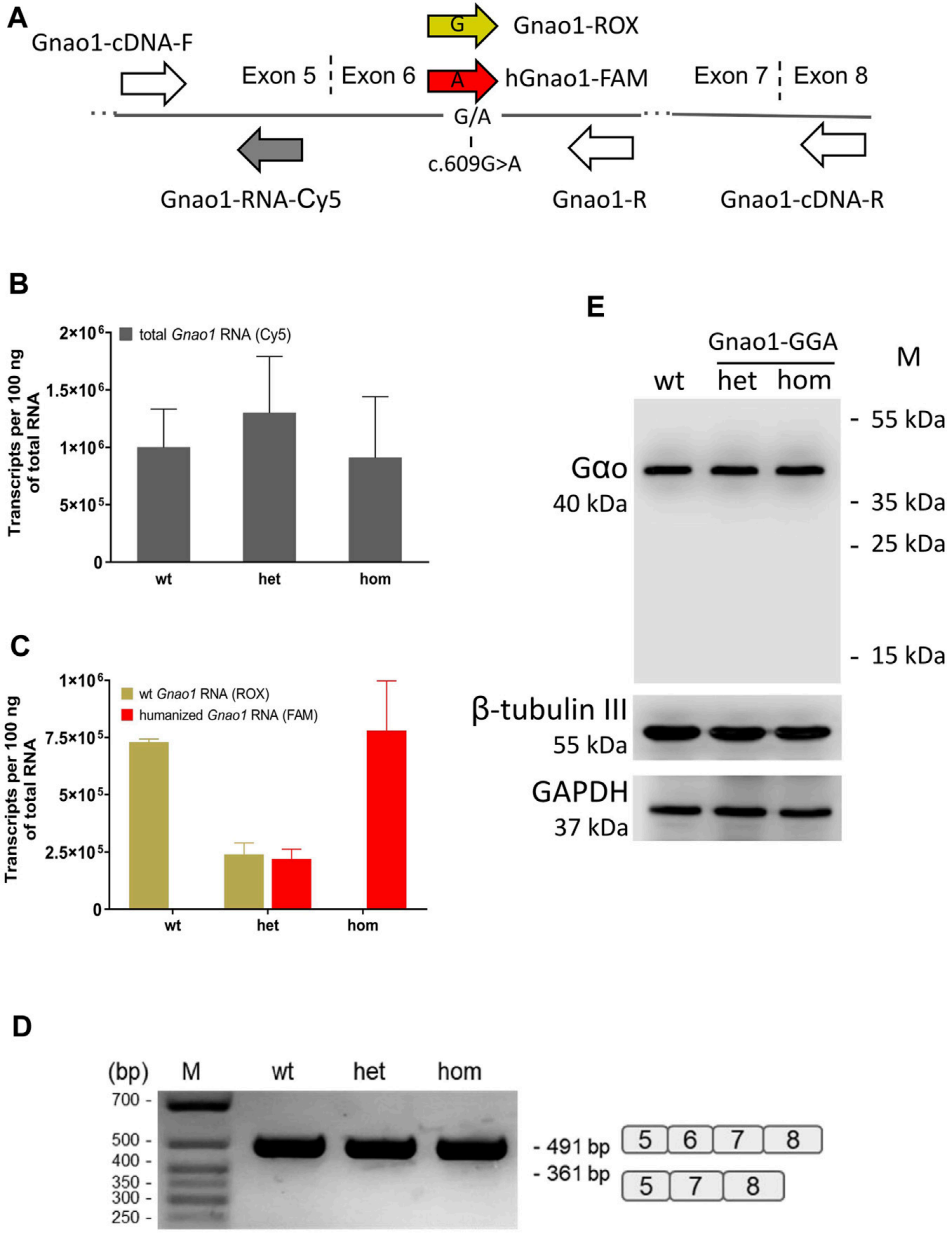
*Gnao1* mRNA and protein expression in the brain of Gnao1-GGA mice. **(A)** Schematically shown the position of primers and probes along *Gnao1* mRNA used for allelic qPCR and exon 6 splicing analysis (see *Materials and Methods*). The vertical dash lines mark the exon junctions. **(B)** The total amount of *Gnao1* transcripts in the brains of F2 littermates was assessed by qPCR (Mean ± SD; n = 3 animals per genotype). **(C)** RNA expression of wild-type (wt) and “humanized” *Gnao1* in the brain assessed by allele-specific qPCR of F2 littermates with the wt, heterozygous (het), and homozygous (hom) Gnao1-GGA genotype (Mean ± SD; n = 3). **(D)** RT-PCR products with primers Gnao1-cDNA-F and -R spanning exons 5 to 8 from representative F2 littermates were analyzed in an agarose gel. A schematic representation of the spliced variants is given to the right of the gel. **(E)** Protein brain extracts from the representative F2 littermates (see Supplementary Figure S6) were analyzed by Western blotting for the occurrence of the truncated forms of the Gαo protein. GAPDH and β-tubulin III are loading controls.

By qPCR, we assessed the expression of *Gnao1* variants in F2 littermates of different genotypes (Figure 3). We adapted *Gnao1*-specific TaqMan probes that were earlier used for genotyping (Figures 2B, C) for mRNA detection (Figure 3A; Supplementary Figure S4). Using a Cy5-labeled probe mapping to exon 5 (Figure 3A), we demonstrated that levels of total *Gnao1* transcripts don’t strongly differ in the wild-type, heterozygous, and homozygous genome-edited mice (n = 3 animals per group) present in the same F2 litter (Figure 3B). Allelic, exon 6-specific probes (Figure 3A) confirmed compositions of the *Gnao1* transcripts: wild-type F2 mice express only murine *Gnao*1 mRNA, homozygous Gnao1-GGA animals contain “humanized” *Gnao1* mRNA, and heterozygous Gnao1-GGA mice express the murine and “humanized” transcripts in the equimolar ratio (Figure 3C). Additionally, we quantified *Gnao1* transcripts in the Gnao1-GGA line and didn’t reveal significant changes compared to the endogenous levels observed in the CBA control (Supplementary Figure S5).

Intronic substitution c.593 + 762C>A introduced into the genome of Gnao1-GGA mice alters polypyrimidine motif of the splice acceptor site with consensus (Y) nNCAGG (Figure 1B). Such modification can potentially decrease efficiency of exon 6 splicing and result in synthesis of transcripts with skipped exon 6. By PCR with primers spanning exons 5-8, we didn’t detect exon 6-skipped *Gnao1* mRNA in the brains of genome-edited mice (Figure 3D). Complementing mRNA data, no truncated forms of Gαo protein (<40 kDa) were detected in Gnao1-GGA mice (Figure 3E). Quantification of the Gαo abundance in the brain of F2 littermates didn’t reveal overt changes (Supplementary Figure S6). Gαo production level in Gnao1-GGA mice is particularly important considering the *Gnao1* role in malignant transformation and tumorigenesis (Liu et al., 2014; Xu et al., 2018; Song et al., 2021). Our data also confirms that GGA doesn’t present a “translationally slow” codon for glycine in mice.

**FIGURE 4:**
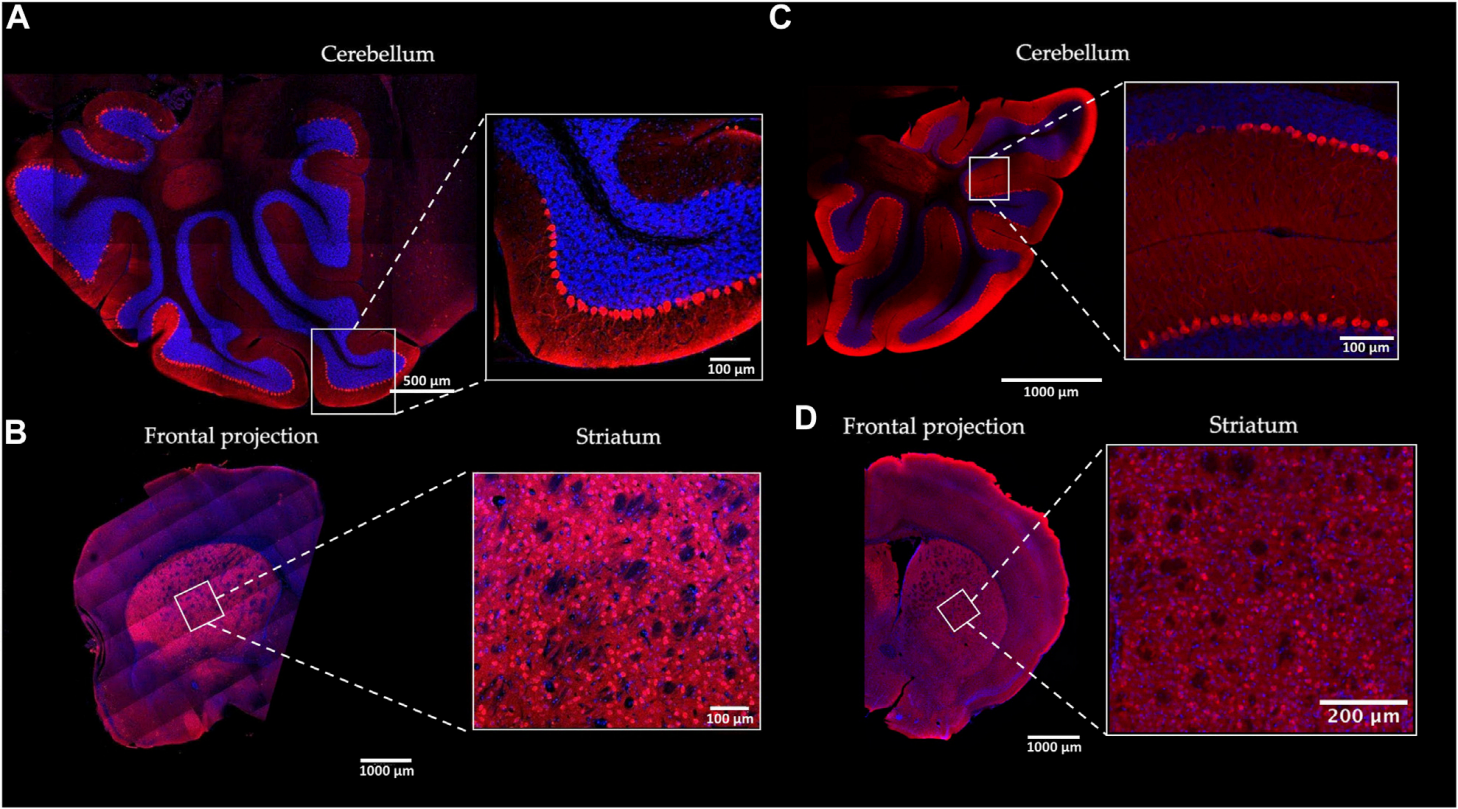
Gαo protein localization in the mouse brain. The distribution pattern of Gαo in the brain of homozygous Gnao1-GGA mice **(A, B)** was compared to the wild-type CBA control mice **(C, D)**. Sagittal (cerebellum) **(A)** and Coronal (striatum) **(B)** cryosections of the Gnao1-GGA brains were examined by immunofluorescence with Gαo-specific antibody (red). The nuclei were stained with DAPI (blue). Similarly, sagittal **(C)** and coronal **(D)** brain cryosections of the CBA mice were prepared and analyzed. Scale bars are shown for each image.

To determine the Gαo expression profile in the brain, we analyzed brain sections of the Gnao1-GGA and the control mice by immunofluorescence with Gαo-specific antibody (Figure 4; Supplementary Figure S7). Our data produced with the wild-type mice (Figures 4C, D; Supplementary Figure S7) is in agreement with the studies on Gαo localization in the rodent brain (Worley et al., 1986; Schüller et al., 2001; Choi et al., 2016; Cha et al., 2019) and confirms the role of the Gαo subunit in the main olfactory epithelium (Wekesa and Anholt, 1999; Cha et al., 2019; Corey et al., 2021) and vomeronasal organ (Berghard and Buck, 1996; Luo et al., 2002), cerebellum (Roldán-Sastre et al., 2021), and striatum (Muntean et al., 2021). Similar profile of the Gαo localization was shown in the brain regions of Gnao1-GGA mice (Figures 4A, B). The most abundant Gαo was in the cerebellar cortex, basal ganglia, and olfactory bulb, while fewer Gαo-positive cells were observed in the layers of the cerebral cortex. In the cerebellum of Gnao1-GGA mice, the Gαo was specifically detected in the neuronal cell bodies (somata) and dendrites of the Purkinje cells (Figure 4A; Supplementary Figure S8) similar to Gαo localization in wild-type mice (Roldán-Sastre et al., 2021).

We conclude that the expression of the “humanized” *Gnao1* in Gnao1-GGA mice is similar to the wild-type mice at the RNA and protein levels. Gαo localization profile in the brain region excludes unintended mosaic changes on the manipulated gene.

### Off-targets and toxicity of *Gnao1*-editing manipulations

A common concern with animal models created using CRISPR/Cas9 is the cleavage of the genomic off-target sites. While whole genome sequencing of the founder mice is preferred to detect unintended genomic modifications, this method is not readily available to all research groups. On the other hand, bioinformatics tools allow *in silico* prediction of the off-target sites (Heigwer et al., 2014) and experimentally verify the most likely modified loci. We checked whether *Gnao1*-unrelated genomic regions were affected in Gnao1-GGA mice. Fifty-three potential off-target sites were predicted by the E-CRISP Evaluation tool (Heigwer et al., 2014) for sgRNA designed to target *Gnao1*, and the top 10 targets are listed in Table 1. With the stringent criteria for the tolerated edit distance (see *Materials and Methods*), off-targets in *Tmem260, Aurkaip1,* and the locus of X-chromosome were predicted (Table 1). All three potential off-targets contained minimum (0–1) PAM-proximal mismatches to the 19-mer sgRNA sequence.

**TABLE 1:** Predicted off-target genome modification with sgRNA directed to *Gnao1*.

Genomic sequence (PAM)	MM*	Locus	Gene	Intron/Exon	Efficacy-score
Target:					
GCT​TTC​CCT​GAC​TCC​CTG​CAGG	—	chr8	*Gnao1* (Gene ID: 14,681)	Intron 5	71,25
Off-target:					
**T**CTT​TCC​CTG​ACT​CCC​TGCTAG	1	chr14	*Tmem260* (Gene ID: 218,989)	Intron 4	19,44
G**GC**TTC​CCT​GAC​TCC​CTG​CTGG	2	chr4	*Aurkaip1* (Gene ID: 66,077)	Exon 3	58,54
** T**CTT​TCC​CTG​ACT​C**A**CTG​CTGG	2	chrX	N/A (NC_000086.8)	N/A	36,14
GCT​TTC​CCT​GA**G**TCC​CTG**A** TGG	2	chr5	*Mmd2* (Gene ID: 75,104)	Intron 2	62,56
GCT​TTC​CCT​GAC​TCC**AG**GCAGG	2	chr5	*Mad1l1* (Gene ID: 17,120)	Intron 17	61,22
GCT**C**T**T**CCT​GAC​TCC​CTG​CAGG	2	chr1	N/A (NC_000067.7)	N/A	61,17
GCT​TTC​CC**C**GAC​TCC​C**A**GCCAG	2	chr9	*Smarca4* (Gene ID: 20,586)	Intron 24	43,50
**A**CTT​TCC​CTG**T**CTC**T**CTG​CAGG	3	chr18	*Greb1l* (Gene ID: 381,157)	Intron 16	47,43
**C**CT**C**TCC​CTG​ACT**G**CCT​GCGGG	3	chr13	*Arl15* (Gene ID: 218,639)	Intron 5	43,39
**T**CTT​TCC​CT**C**ACT**G**CCT​GCAGG	3	chr 5	*Plb1* (Gene ID: 665,270)	Intron 1	42,75

*Number of mismatches (MM) between sgRNA and the genomic target. Mismatched nucleotides are marked in bold.

**FIGURE 5:**
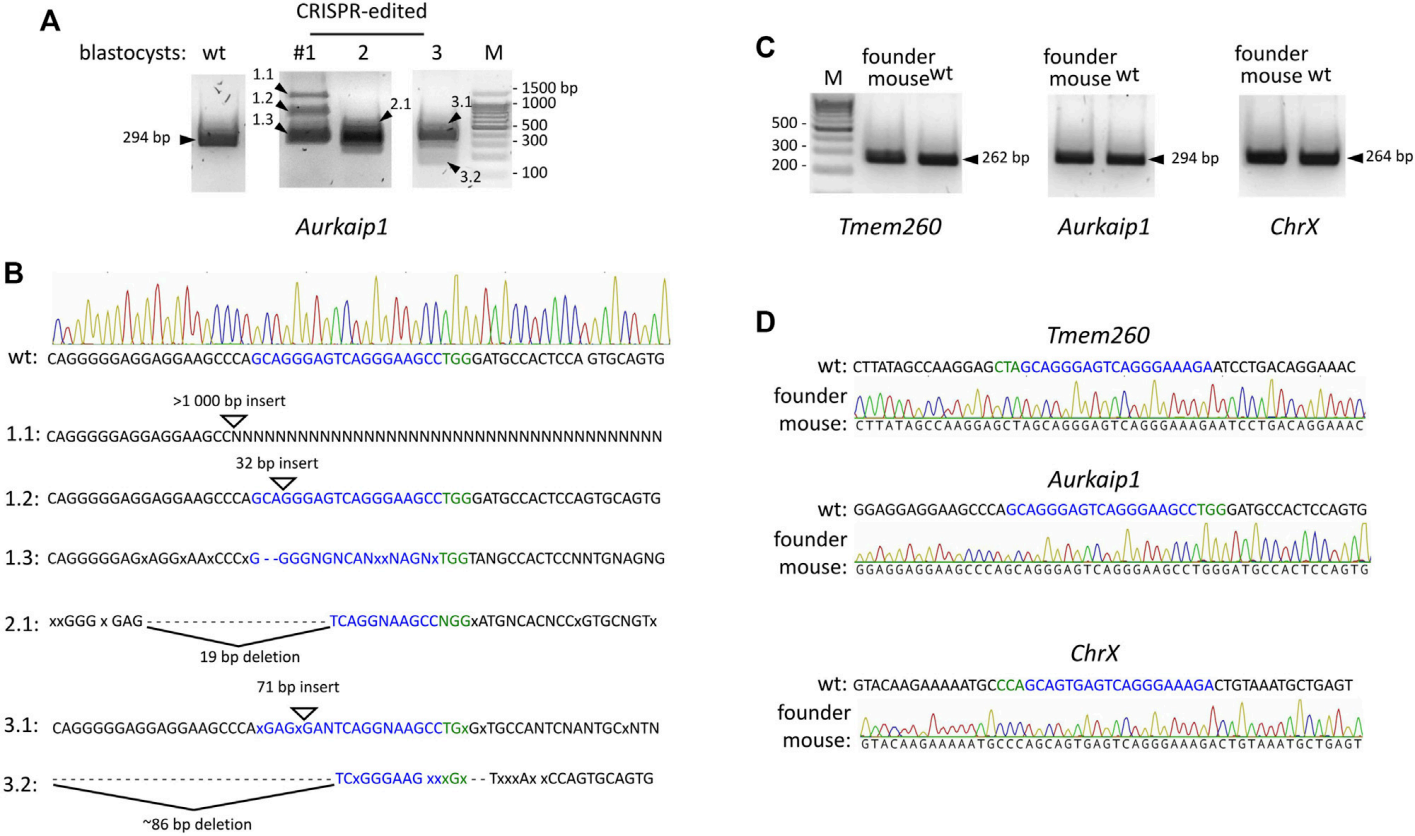
CRISPR/Cas9 off-target activity in microinjected embryos and genome-edited Gnao1-GGA mice. **(A)** Off-target modifications in the *Aurkaip1* coding region analyzed in the microinjected (#1–3) and untreated, wild-type blastocysts. The *Aurkaip1* genomic region with the putative off-target site was amplified by PCR, and amplicons were analyzed in agarose gel. Amplicons of various sizes (marked by arrowheads) were subjected to Sanger sequencing. **(B)** Interpretation of the histograms revealed the mosaic nature of the sequences. Presented are the alternative to wild-type allele sequences characterized by indels. sgRNA off-target sequence and PAM are shown in blue and green respectively, mismatches are shown as “x,” deletions as “-,” unspecified nucleotides as “N.” **(C)** Predicted off-target sites were analyzed in the CRISPR-edited genomic material from the founder Gnao1-GGA mice. Genomic regions encompassing off-targets in *Aurkaip1* (294 bp), *Tmem* (262 bp), and *ChrX* (264 bp) were amplified and analyzed in agarose gel. A single band of the expected size was detected for each amplicon. **(D)** Absence of off-target cleavage in founder Gnao1-GGA mice was confirmed by Sanger sequencing of amplicons from *Aurkaip1, Tmem,* and ChrX regions.

Of particular interest was the off-target in the coding region of the *Aurkaip1* gene. The *Aurkaip1* gene encodes aurora kinase A interacting protein, which is involved in the cell cycle progression (Kiat et al., 2002; Lim and Gopalan, 2007). In the blastocyst assay, we confirmed the off-target activity of the CRISPR/Cas9 complex, and formation of indels in *Aurkaip1* was revealed by analysis of the PCR amplicons in agarose gel and Sanger sequencing (Figures 5A, B, respectively). The analyzed blastocysts were mosaics and, in addition to the wild-type allele, contained single nucleotide mutations at the cut site, as well as extended indels (commonly <100 bp, but also >1,000 bp long). Disruption of *Aurkaip1* could potentially contribute to the low survival rate of the CRISPR-edited embryos in our experiments. Indeed, viability of the Aurkaip1 KO mice was previously assessed and characterized by embryonic lethality prior to organogenesis (IMPC, 2022). Importantly, we didn’t detect disruption of the *Aurkaip1* in the genomic material of the founder CRISPR-edited mouse (Figures 5C, D). The off-target modification of the *Tmem260* and chromosome X locus was also not detected (Figures 5C, D). The remaining predicted off-targets were located within non-coding genomic regions (Table 1) or contained more than one mismatch in the PAM-proximal region which contributes to cleavage efficacy. Therefore, it is less likely that changes in these areas can lead to dysfunction of the corresponding genes. Confirming the absence of the adverse effects of the genome editing, no neuropathological changes in Gαo1-rich structures were revealed by histological examination of the Gnao1-GGA mice brain sections (Supplementary Figure S9).

We conclude that the reported genome-edited mice with the “humanized” fragment of the *Gnao1* exon 6 provide a suitable model for preclinical safety studies of RNA-targeting therapeutics for the c.607G>A variant of GNAO1-associated encephalopathy.

## Discussion

Personalized medicine holds a huge promise for severe genetic diseases (Kim et al., 2019; Wang et al., 2020; Diener et al., 2022), and requires the appropriate animal models (Kalmykov et al., 2018; Aartsma-Rus and van Putten, 2019; Polikarpova et al., 2022; Vázquez-Domínguez and Garanto, 2022). Sequence-specific RNA-based drugs, such as antisense oligonucleotides (ASO) and RNAi therapeutics (Zhu et al., 2022; Zogg et al., 2022), are currently in development as a new generation of medicine to treat dominant neurological disorders such as Huntington’s disease, amyotrophic lateral sclerosis, spinocerebellar ataxia, and several others (Zhao et al., 2017; Iannitti et al., 2018; Miniarikova et al., 2018; Southwell et al., 2018; Martier et al., 2019; Aimiuwu et al., 2020). Humanized mouse models are in demand to facilitate safety and efficacy studies of such innovative classes of drugs (Nair et al., 2019; Zhu et al., 2019; Vázquez-Domínguez and Garanto, 2022). For instance, several humanized mouse models of Huntington’s disease are currently available for the preclinical evaluation of SNP-dependent ASO and RNAi-based gene therapy (Miniarikova et al., 2018; Southwell et al., 2018). Full humanization of the mouse gene, including the replacement of coding and non-coding sequences with human sequences (Zhu et al., 2019), is desired but isn’t a strict requirement. Partial humanization, such as replacing a small gene fragment or individual base pairs at a critical location, may be beneficial for testing RNA-based drugs.

Here we report a mouse model Gnao1-GGA (Bardina et al., 2021) with the 68-nucleotide coding fragment of the endogenous *Gnao1*, spanning the junction of exons 5 and 6, 100% identical to the wild-type human sequence (Supplementary Figure S3). To locally “humanize” highly conserved *Gnao1*, it was sufficient to introduce a single synonymous substitution c.609 G>A into Gly203-coding triplet using CRISPR/Cas9 technology (Figure 1). Additional mutation c.593 + 762C>A was introduced into the intronic sequence for mice genotyping (Figure 2). Generated Gnao1-GGA mice are useful to address the safety of the RNA-based drugs for the orphan disease GNAO1 encephalopathy caused by dominant variant c.607 G>A. Antisense therapy (Zatsepin, 2020) and vectorized RNAi therapeutics (Luchkina et al., 2021) are currently in development for this severe disorder. Both types of drugs act similarly by binding in a sequence-specific manner to the mutation site in the *GNAO1* mRNA and preventing the synthesis of the protein with dominant mutation. Expected that production of the functional Gαo protein will not be affected due to mismatches of RNA drugs with the wild-type *GNAO1*. Unintended effects on the wild-type *GNAO1 in vivo* can be assessed in the Gnao1-GGA mice with a “humanized” target sequence for RNA therapeutics in the exon 6. A pilot ASO safety study was announced at the second GNAO1 European Conference streamed online on 1–3 October 2020 (Zatsepin, 2020). RNA-based therapies are by no means a universal solution for all patients with GNAO1 encephalopathy. This sequence-specific approach might be helpful only for gain-of-function or dominant-negative variants with severe phenotypes. Patients with the loss-of-function mutations and strong evidence of the GNAO1-haploinsufficiency (Feng et al., 2017; Muntean et al., 2021; Krenn et al., 2022; Lasa-Aranzasti et al., 2022) will not benefit from the gene suppressing RNA therapeutics, instead, gene replacement therapies should be considered.

Our GNAO1-GGA mouse line expands the list of reported animal models with the *Gnao1* knock-ins. The first mouse model of *GNAO1*-related epilepsy was created using genetically modified embryonic stem cells with G184S mutation (Kehrl et al., 2014). Following studies utilized CRISPR/Cas9-based methods for precise genome editing. Heterozygous substitutions R209H and C215Y allowed recapitulating movement disorder phenotype in mice (Larrivee et al., 2020; Silachev et al., 2022). Feng et al. (2019) reported successful targeting of the *Gnao1* exon 6 by CRISPR/Cas9 and generation of mice with G203R. However, the follow-up study showed that genome editing resulted in the unintended mutation of the splice site that disrupted the expression of the edited *Gnao1* allele (Feng et al., 2022). This case clearly demonstrates the absolute requirement for detailed expression analysis of the targeted gene following editing procedures. Invertebrate models of GNAO1 encephalopathy were generated by introducing orthologous mutations G42R, G203R, and R209C into the *goa-1* gene o*f C. elegans* (Wang et al., 2021). Finally, a line of *Drosophila melanogaster* with humanized *gnao1* exons 2-3 and 4-7 was developed. In the resulting model, the amino acid sequence of the Gαo corresponds to the human ortholog, but the nucleotide sequence of the gene is different (Savitsky et al., 2020).

**FIGURE 6:**
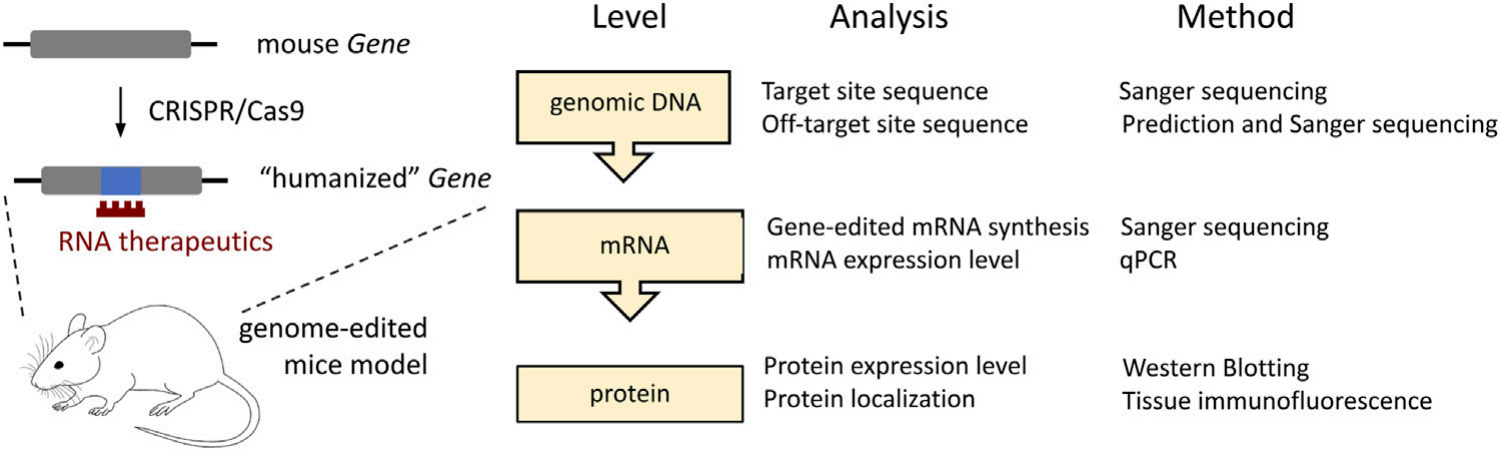
The minimal requirement for the gene analysis following CRISPR/Cas9-mediated editing of the protein-coding targets in mice. In the current studies, such analysis is applied to the Gnao1-GGA mice.

Our work on Gnao1-GGA mice stands out from similar studies by scrutinized analysis of the edited gene at the genomic DNA, mRNA, and protein levels (Figure 6). Despite minor modifications of endogenous *Gnao1* (Figure 2D), two single-base substitutions can potentially cause an imbalance in allele expression. In particular, intronic mutation c.593 + 762C>A is located within the splicing signal and can affect the pre-mRNA processing leading to skipping of exon 6 (Aoyama et al., 2017). We did not detect transcripts with skipped exon 6 in Gnao1-GGA mice (Figure 3D). Moreover, we demonstrated that the level of the murine wild-type and CRISPR-edited *Gnao1* transcripts is comparable in control and Gnao1-GGA mice, respectively (Supplementary Figure S5). Using a Gαo-specific antibody, we confirmed that neither Gαo protein level (Supplementary Figure S6) nor localization in the specific brain areas (Figures 4; Supplementary Figure S8) was affected in CRISPR-treated animals. While the off-target activity of CRISPR/Cas9 complexes was shown in blastocysts (Figure 5А, B), no modifications of the predicted off-target sites were detected in the founder mouse using simple PCR-based techniques and Sanger sequencing (Figures 5C, D).

To conclude, humanization of the mouse genome is in high demand. Increasing the efficiency and reducing the off-target activity of CRISPR/Cas9 technology (Han et al., 2020) will allow routine humanization of individual codons (Zhu et al., 2019) and aid the generation of mouse models with human clinical variants ∼50% of which are presented by point mutations (Rees and Liu, 2018). Current studies contribute to this field and can serve as a guide to how cost-effective methods available in each laboratory can be used to verify target gene expression following even minor CRISPR/Cas9-mediated genome modifications (Figure 6). In the near future, technological advances in mouse genome engineering will accelerate the testing of innovative drugs for severe genetic disorders and make such therapies available for patients.

## Data Availability

The original contributions presented in the study are included in the article/Supplementary Materials, further inquiries can be directed to the corresponding author.
