# Supplementary material for: CRISPR/Cas9-generated mouse model with humanizing single-base substitution in the Gnao1 for safety studies of RNA therapeutics
Source: Front Genome Ed. 2023 Apr 3;5:1034720. doi: 10.3389/fgeed.2023.1034720 (PMC10106585; doi:10.3389/fgeed.2023.1034720)
Supplement: Supplementary file 1 [file DataSheet1.pdf]

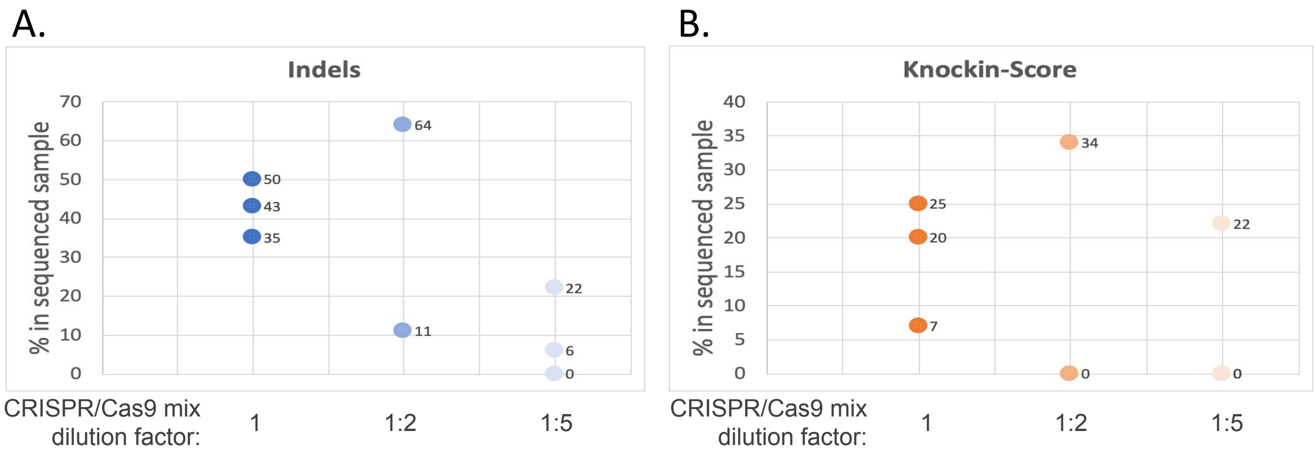

Figure S1. The efficiency of CRISPR/Cas-mediated genome editing of the *Gnao1* locus in mice embryos. Genome editing mix (50 ng/uL Cas9 mRNA, 18.6 ng/uL sgRNA, 10 uM ssODN) was microinjected into two- pronuclear mouse zygotes undiluted (dilution factor 1) or at 2- and 5-fold diluted concentrations. % of indels (A) and knockin-score (B) was evaluated for 2-3 single blastocysts from each group by Sanger sequencing. Reads were analyzed with the online tool Syntego ICE Analysis. Each dot on the graphs represents data from a single blastocyst. The dilution of the editing components resulted in a decrease in the number of indels (which reflects the drop in the cutting efficiency) and a decrease in the knockin-score (which corresponds to the reduction of the homologous recombination efficiency).

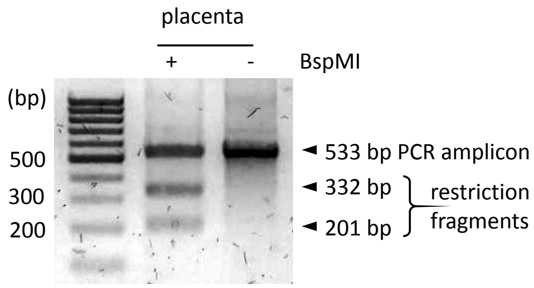

Figure S2. Knock-in detection in the placenta material of the CRISPR/Cas9-edited mouse. Genomic DNA was extracted from the placenta of P0 pup and a 533 bp PCR amplicon was produced. Digestion of the amplicon into 332 bp and 210 bp fragments reveals the presence of the BspMI restriction site and suggests ssODN-mediated knock-in in the CRISPR/Cas9-edited mouse. Incomplete digestion of the amplicon is due to the presence of the genetic material from the recipient mice and/or results from the mosaic pattern of genome editing.

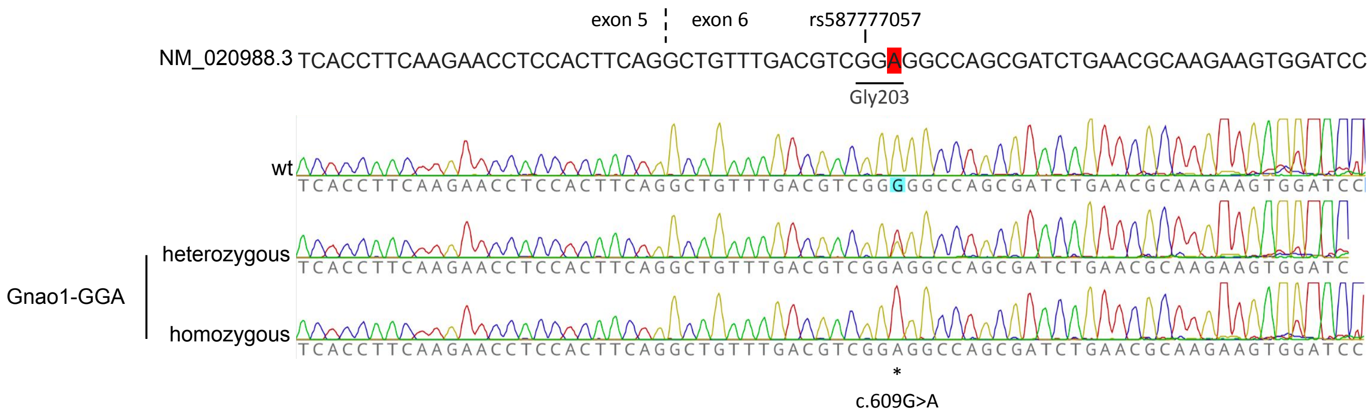

Figure S3. *Gnao1* mRNA sequence in the wild-type, heterozygous and homozygous Gnao1-GGA mice. Representative chromatograms of the Gnao1 mRNA from mice with different genotypes are aligned to the sequence of the human GNAO1 transcript (NM_020988.3). Vertical dash line marks junction of exons 5 and 6.

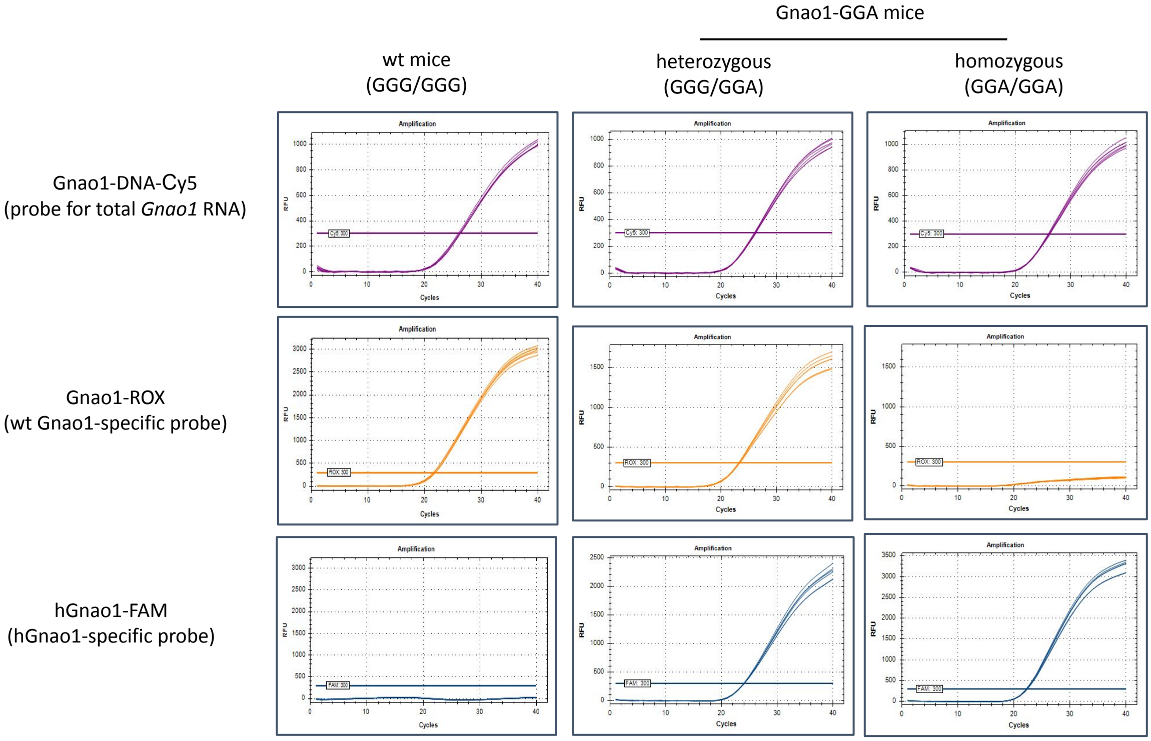

Figure S4. Detection of the wild-type and humanized *Gnao1* transcripts by allelic qPCR with specific probes. Representative amplification curves for homozygous and heterozygous Gnao1-GGA mice as well as wild-type mice are shown. In brackets the Gly203-coding triplet for each type of mice is specified. Data confirms the specificity of the allelic qPCR probes and demonstrates the expression of the *Gnao1* mRNA variants.

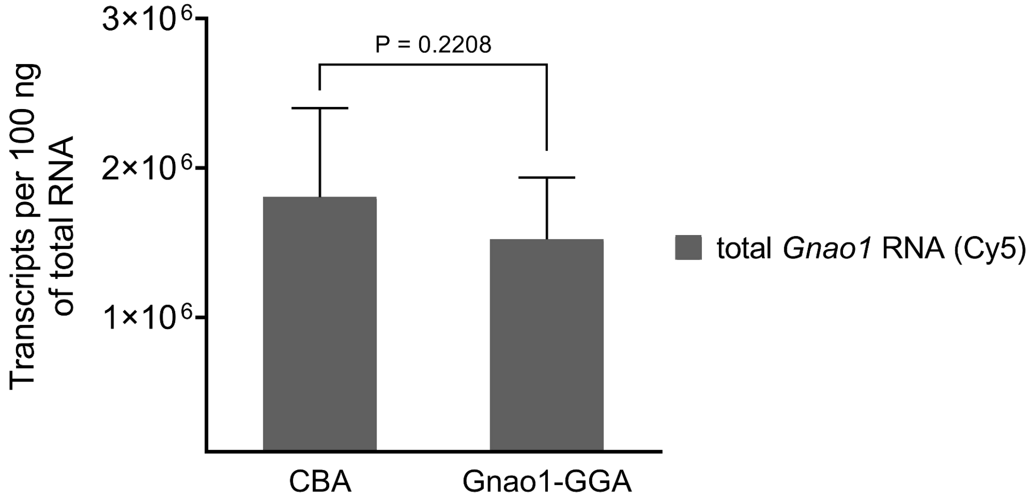

Figure S5. The total amount of Gnao1 transcripts in the brains of adult wild-type CBA and Gnao1-GGA lines. Analysis by qPCR (Mean±SD; n=6). P-value was calculated with the Mann–Whitney test.

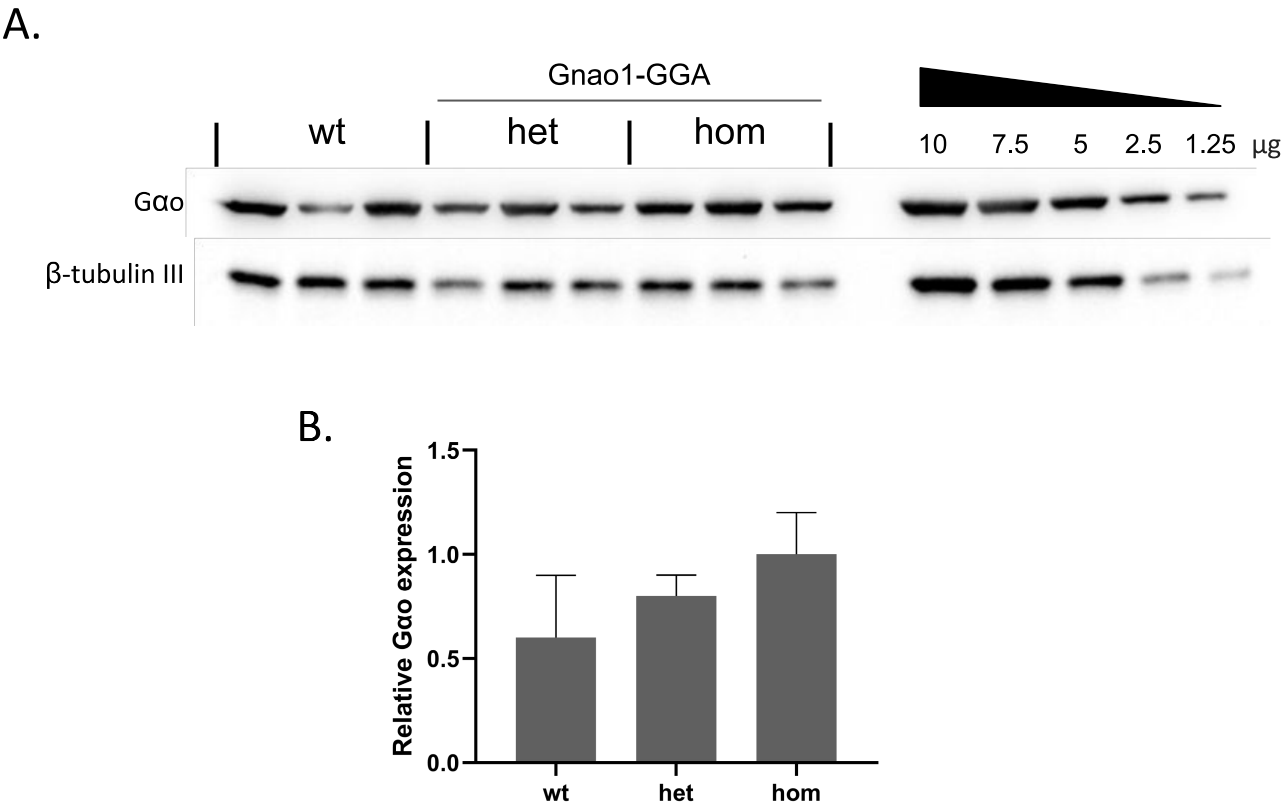

Figure S6. The relative abundance of the Gαo protein in the brain of CRISPR-edited mice. A. Brain extracts from F2 littermates with the wild-type, hetero-, and homozygous Gnao1-GGA genotypes (same as in Figure 3b-c) were analyzed by Western Blotting. β-tubulin III is a loading control. On the right side of the membrane are serial dilutions of the control brain homogenate (10, 7.5, 5, 2.5, 1.25 µg of total protein per lane). The protein dilutions demonstrate the working range of Gαo- and β-tubulin III-specific antibodies. B. The Gαo protein bands were quantified using ImageJ and normalized to β-tubulin III (Mean±SD; n=3 animals per each group).

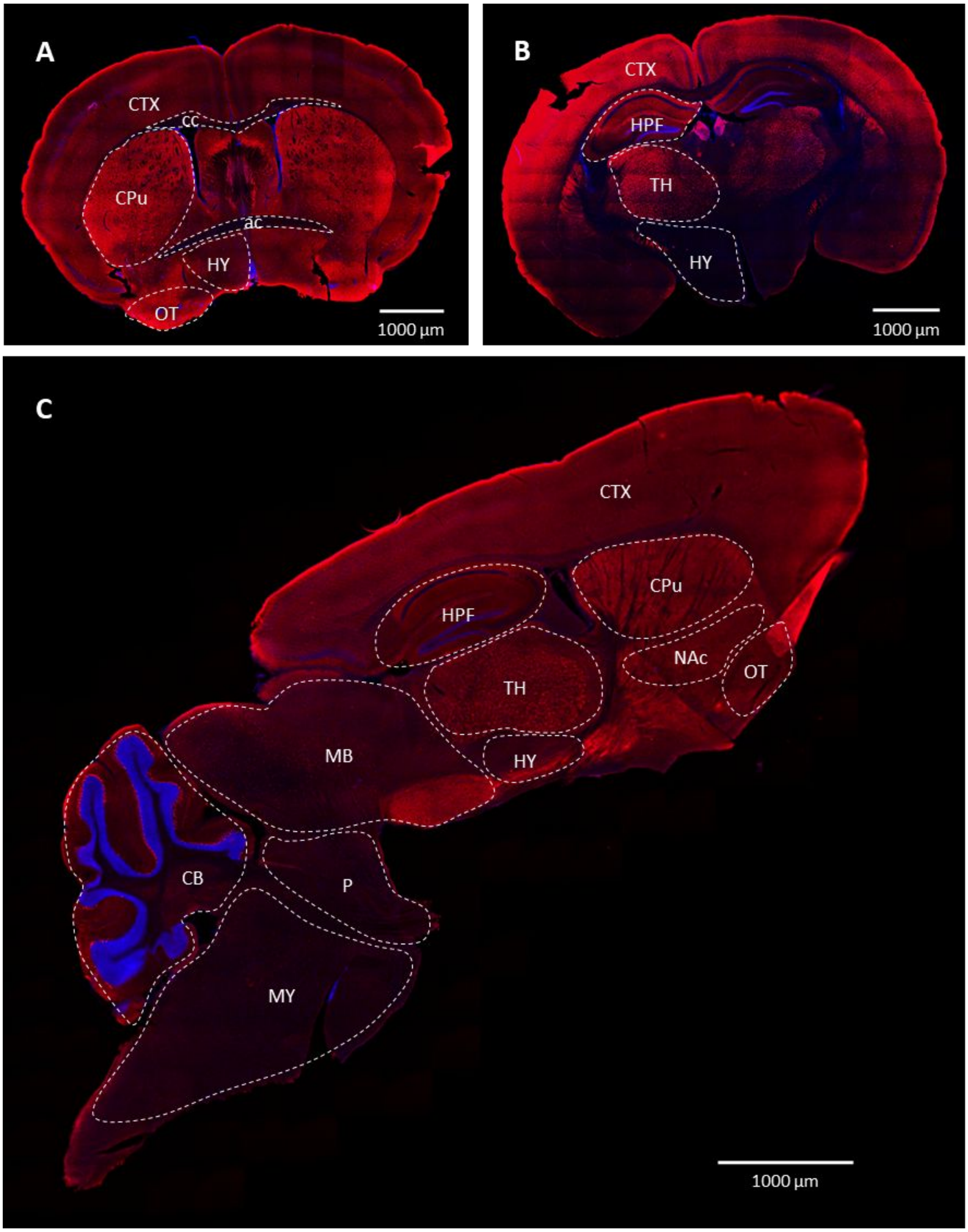

Figure S7. Gαo localization in the brain of the wild-type mice. Anesthetized mice were subjected to cardiac perfusion with 10% buffered formalin to obtain brain preparations. After isolation, the brain samples were incubated in formalin for 24 hours. Coronal (A, B) and sagittal (C) sections of the fixed brain were sliced on the vibratome Microm HM-650V (Thermo Fisher). The brain sections were stained with anti-Gαo antibody (rabbit; Thermo Fisher Scientific, PA5-30044), followed by staining with Alexa Fluor 633 anti-rabbit secondary antibody (Invitrogen, A21072) (red). The nuclei were counterstained with DAPI dye (blue). Images were acquired using Zeiss LSM880 confocal microscope equipped with Plan-Apochromat 20x/0.8 M27 objective. CTX - cerebral cortex, CPu - caudoputamen, OT - olfactory tubercle, HY - hypothalamus, cc - corpus callosum, ac - anterior commissure, HPF - hippocampal formation, TH - thalamus, NAc - nucleus accumbens, MB - midbrain, CB - cerebellum, P - pons, MY - medulla.

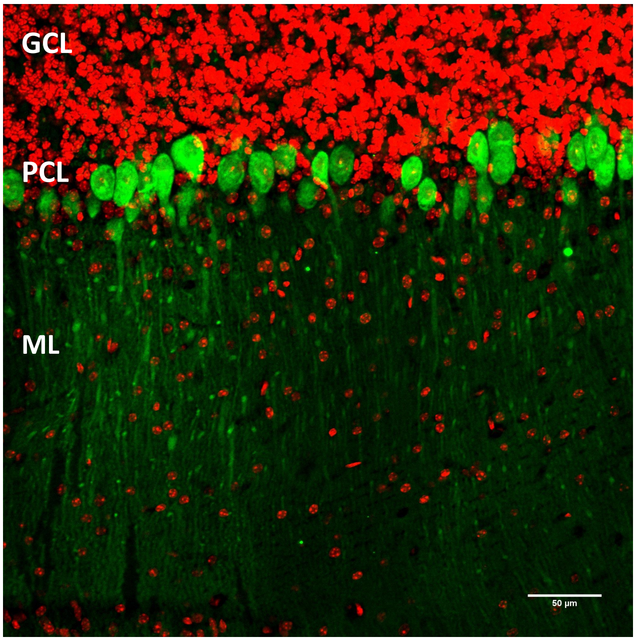

Figure S8. Localization of Gαo (green) in Purkinje cells of cerebellar cortex of Gnao1-GGA mice. Three distinct layers of the cerebellar cortex are marked: the granule cell layer (GCL), the Purkinje cell layer (PCL), and the molecular layer (ML). The nuclei were stained with DAPI (red). 60x objective, scale bar = 50 µm

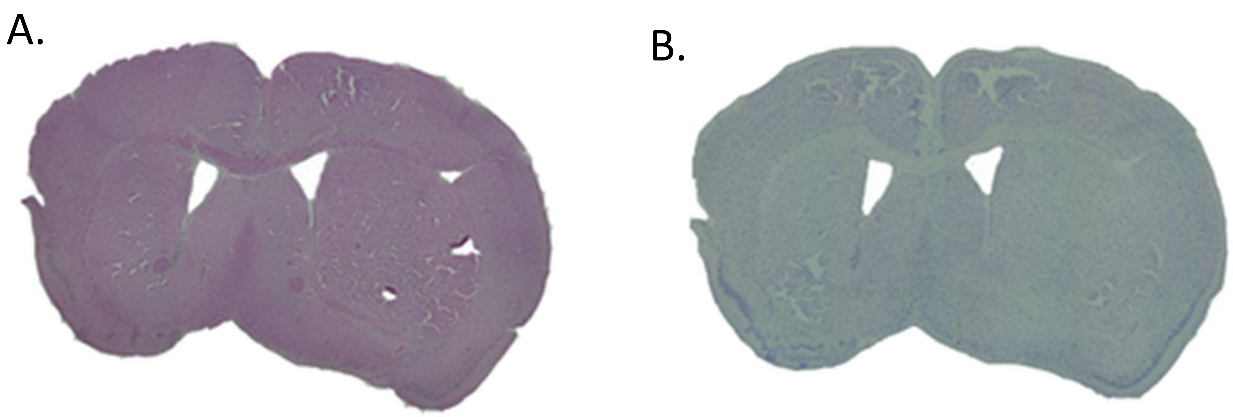

Figure S9. Histological analysis of the frontal brain sections of Gnao1-GGA mice by hematoxylin/eosin (A) and Nissl (B) staining. The figure shows a representative images.
